# Exploring the Link Between Genetic Predictors of Systemic Lupus Erythematosus and Epstein–Barr Virus Infections

**DOI:** 10.1111/1756-185X.70067

**Published:** 2025-02-03

**Authors:** Yanying Piao, Lei Li, Rongxian An, Dinglu Cui, Xinying Cui, Long Jiang, Jingchun Jin

**Affiliations:** ^1^ Depatment of Immunology Yanbian University Hospital Yanji China

1

Systemic lupus erythematosus (SLE) is one of the most common autoimmune diseases (ADs), has widespread clinical manifestations and a chronic relapsing–remitting course, and has different clinical courses and prognoses [1, 2]. The published data showed that AD patients have an increased danger of developing malignancies. According to relevant literature reports, viruses related to SLE include EBV, Parvovirus B19 (B19V), Retro viruses (RVs), Human Endogenous Retroviruses (HERV), Human Immunodeficiency Virus (HIV), Torque Teno Virus (TTV) and Cytomegalovirus (CMV) [3].

Epstein–Barr virus (EBV) is a lymphotripic virus, also known as Human Herpesvirus (HHV) 4 [4]. It is also considered as the most common human viruses and infects at least 90% of adults worldwide [5]. EBV is believed to be associated with the onset of a lot of ADs, such as rheumatoid arthritis (RA), multiple sclerosis (MS), and SLE [6, 7, 8].

Mendelian randomization (MR) analysis is a new‐type epidemiological analysis method that uses instrumental variables (IVs) to analyze genetic variation and assess the causal association between exposures and outcomes [9]. The relevant IVs for MR research are from genome‐wide association studies (GWAS).

The key point in conducting MR analysis was to select proper genetic variants from the open obtainable GWAS database as effective IVs. We selected single nucleotide polymorphisms (SNPs) from the IEU GWAS database (https://gwas.mrcieu.ac.uk/) and FinnGen database (http://www.finngen.fi) for IVs of all exposures, mediators, and outcomes. Instrumental SNPs were related with SLE and cancers at genome‐wide relevance (*p* < 5e‐8). Obtain genome‐wide significant (*p* < 1e‐5) IVs from five EBV infection databases to improve inference and computational abilities. Summary of data in this MR study can be seen in Table S1.

To make sure the facticity and correctness of the inferences regarding the causal relationship between EBV infections and SLE, we have implemented a series of quality control measures to select qualified IVs. We extracted genome‐wide significantly correlated SNPs from the GWAS summary data, with linkage disequilibrium (LD) and independent (*r* ^2^ = 0.001, kb = 10 000) SNPs for sorting. We followed the above steps to screen EBV infections, cancers, and SLE‐related SNPs with significant correlation, linkage equilibrium, and independence from the GWAS database for MR analysis. We also estimated the *F*‐statistic of SNPs to evaluate the strength of instrumental variables. If *F* > 10, it means that if the likelihood of instrumental variable bias is small, SNPs with *F*‐statistic less than 10 should be removed.

In our study, the main statistical analysis was performed to use the R packages (version 4.3.2). Further, we used the “TwoSampleMR” (version 0.5.8) and “MR‐PRESSO” (version 1.0) packages to conduct the MR analysis.

First, we conducted MR forward analysis using SLE as the exposures and anti‐EBV IgG as the outcomes. Then, the genetic instruments selected for the five phenotypes of EBV were used as IVs, and SLE was used as the outcome of reverse MR. We used inverse‐variance weighted (IVW), MR‐Egger, weighted median (WM), weighted mode, and simple mode method for MR analysis to calculate the causality between SLE and EBV infections. MR analysis minimizes the impact of confusion and reverse causal bias. In MR analysis, IVW is the standard approach for summing‐up data and is the primary analysis method [10].

MR Pleiotropy RESidual Sum and Outlier (MR‐PRESSO) was used to further test the level of validity of the MR analysis results, and heterogeneity testing was conducted using MR Egger intercept [11]. We used Cochran *Q*‐test to evaluate data heterogeneity. While *p* < 0.05, it indicates significant heterogeneity, and then using a random effects model with IVW method to infer causal relationship. Pleiotropy refers to the phenomenon where a single locus impacts on various phenotypes. We conducted the MR‐Egger intercept test to appraise and regulate for horizontal pletropy, if *p* > 0.05, it implied that there is no horizontal pleiotropy, in addition to prove the stability of the research results. The results were reported as having corresponding OR and 95% CI, with *p* < 0.05 indicating statistical significance.

We passed through the two‐step Mendelian randomization (TSMR) method, and performed four steps to mediation analysis. Step1: SLE to EBV (total effect β_all). Step2: EBV to SLE. Step3: SLE to cancers (βa). Step4: cancers to EBV (βb). Mediated effect βc: βa × βb. Direct effect β_dir: β_all‐βa × βb.

First, select the appropriate SNP. After eliminating SNPs that were considered insignificant for both exposure and outcome, the resulting SNP sets were merged to obtain IVs for Mendelian randomization analysis.(Tables S2 and S3).

MR analysis results indicated that there was no causal association between the risk of SLE and EBV infections. IVW results suggested that there was no evidence for the effect between the two (anti‐EBV IgG: *p* = 0.074; VCA IgG: *p* = 0.863; EBNA IgG: *p* = 0.358; EA IgG: *p* = 0.993). Further, the MR‐Egger and WM results also proved that there was no causal association between SLE and EBV (Figure 1, Table S4).

**FIGURE 1:**
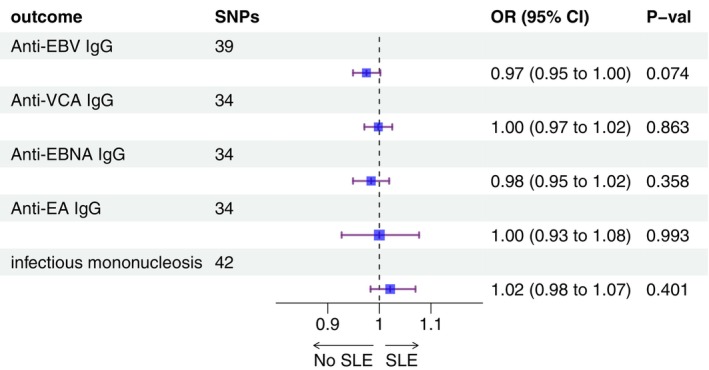
The forest plot of existing causal effect of SLE on EBV.

Furthermore, when EBV infections were used as exposure, we discussed the impact of the causal effect with SLE. The IVW method suggested no significant evidence for casual relationship between EBV infections and SLE. Inefficient discoveries were validated in the evaluation by MR‐Egger and WM method (Figure 2, Table S5).

**FIGURE 2:**
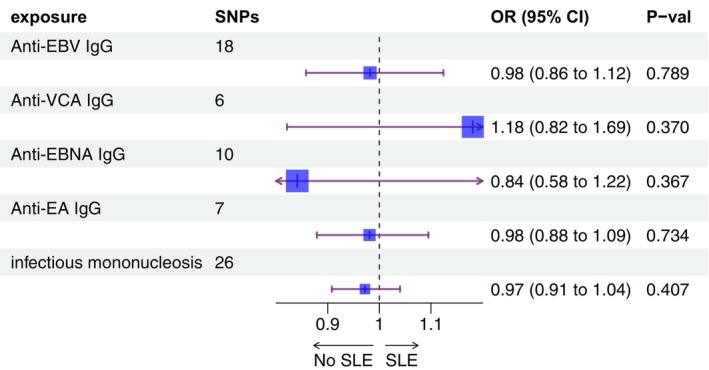
The forest plot of existing causal effect of EBV on SLE.

In order to remove potential impacts on the MR assumption, we further conducted multiple sensitivity analyses to verify whether heterogeneity and pleiotropy in the tested genetic tools would bias the MR results. According to the MR‐Egger intercept analysis, the *p* values are all greater than 0.05, indicating that there is no horizontal diversity in the results, what means that the MR result analysis is effective (Tables S6 and S7).

Finally, to investigate whether cancers affect the relationship between SLE and EBV infection, further mediation analysis will be conducted. The result indicated that the five types of cancers did not affect the causal relationship between the two, and further enhance the credibility of the above results (Table S8).

For a long time, EBV has been considered as a potential trigger of SLE. A large amount of evidence suggests that viruses may be an important environmental factor in the nosogenesis of autoimmune rheumatic diseases [12], especially EBV infection, is closely related to SLE [13]. In our study, we used MR analysis to evaluate the causal association between EBV and SLE. Unexpectedly, our study indicated that there is no support for the relevance of EBV on the increased risk of SLE.

A few studies indicated that patients with SLE have an increased risk of EBV infection. An epidemiological survey in Qingdao, China, showed that SLE patients have an obviously higher danger of severe EBV infection in relation to the general population [14]. An observational study also demonstrated that SLE patients had been susceptible to viral infections, including the EBV infections [15]. In another observational study, the reactivities of IgG to EBV antigens (EBNA1) in SLE serum samples were measured, indicating that EBV EA/D IgG was significantly increase in SLE [4]. In summary, a strong correlation was found between SLE and EBV infection. However, according to our MR analysis, there is no related links between SLE and EBV infection.

Much serological evidence suggests a correlation between EBV infection and SLE. However, our reverse MR analysis shows no significant causal relationship between EBV infection and SLE, indicating that the small sample size might have influenced the results.

MR is a powerful means for studying and clarifying the causal relationship between exposure and outcomes. Currently, there are no MR studies reported on EBV infection and SLE. Our study has several important advantages. First, utilize the aggregated statistical data of the five major diseases to expand the list of EBV infections. Second, we used large‐scale GWAS genetic data to study the causal association between EBV‐related antibody titers in serum and SLE, improving statistical efficacy. Third, large sample sizes and robust SNPs can help detect causal effects with high precision. Forth, we conducted mediation analysis to further exclude the influence of cancers on the results of our study.

However, our study still has some shortcomings. Above all, the IVs used mostly came from the European population; therefore, the results in our article may not apply to other populations. Then, there is heterogeneity in the analysis results, as the GWAS data used were not grouped, making it impossible to proof whether there is a causal association between EBV infection and SLE in individuals of different genders, physical conditions, and age groups, such as, whether adults and elderly people are at risk of SLE when infected with EBV. Finally, as our IVs were selected based on a relaxed significance threshold of 1e‐05 instead of the classical 5e‐08 to ensure that all study participants had IVs, confounding factors appeared in our results.

A lot of research has suggested a potential causal relationship between SLE and EBV infections, but according to our study using Mendelian randomization in this article, there is no causal relationship between EBV infections and genetically predicted SLE. Further follow‐up studies may be needed to determine whether the results are influenced by factors such as age and gender in order to obtain more accurate conclusions.

## Author Contributions

**Yanying Piao:** writing – original draft, writing – review and editing, methodology, formal analysis, visualization, conceptualization. **Lei Li and Rongxian An:** writing – review and editing, visualization, methodology, formal analysis. **Xinying Cui** and **Long Jiang:** software, formal analysis, data curation. **Dinglu Cui:** supervision, data curation, conceptualization. **Jingchun Jin:** supervision, funding acquisition, data curation, conceptualization.

## Ethics Statement

The datasets generated during and/or analyzed during the current study are publicly available. Ethical review and approval were not required for the study on human participants in accordance with the local legislation and institutional requirements.

## Consent

The authors have nothing to report.

## Conflicts of Interest

The authors declare no conflicts of interest.

## Supporting information


Table S1.
Table S2.
Table S3.
Table S4.
Table S5.
Table S6.
Table S7.
Table S8.

## Data Availability

All data are available upon request.

## References

[1] S. Lazar and J. M. Kahlenberg , “Systemic Lupus Erythematosus: New Diagnostic and Therapeutic Approaches,” Annual Review of Medicine 74 (2023): 339–352, 10.1146/annurev-med-043021-032611. 35804480

[2] D. Zucchi , E. Silvagni , E. Elefante , et al., “Systemic Lupus Erythematosus: One Year in Review 2023,” Clinical and Experimental Rheumatology 41, no. 5 (2023): 997–1008, 10.55563/clinexprheumatol/4uc7e8. 37133502

[3] M. Quaglia , G. Merlotti , M. De Andrea , C. Borgogna , and V. Cantaluppi , “Viral Infections and Systemic Lupus Erythematosus: New Players in an Old Story,” Viruses 13, no. 2 (2021): 277, 10.3390/v13020277. 33670195 PMC7916951

[4] J. Knudsen , N. H. Trier , A. H. Draborg , et al., “Elevated Antibody Titers to Epstein–Barr Virus and Cytomegalovirus in Patients with Drug‐Induced Lupus,” Viruses 15, no. 4 (2023): 986, 10.3390/v15040986. 37112967 PMC10144390

[5] G. de‐Thé , N. E. Day , A. Geser , et al., “Sero‐Epidemiology of the Epstein–Barr Virus: Preliminary Analysis of an International Study—A Review,” IARC Scientific Publications (1971) 11 Pt 2 (1975): 3–16. 191375

[6] A. Draborg , J. M. Izarzugaza , and G. Houen , “How Compelling are the Data for Epstein–Barr Virus Being a Trigger for Systemic Lupus and Other Autoimmune Diseases?,” Current Opinion in Rheumatology 28, no. 4 (2016): 398–404, 10.1097/BOR.0000000000000289. 26986247

[7] S. S. Soldan and P. M. Lieberman , “Epstein–Barr Virus and Multiple Sclerosis,” Nature Reviews. Microbiology 21, no. 1 (2023): 51–64, 10.1038/s41579-022-00770-5. 35931816 PMC9362539

[8] N. H. Trier , B. E. Holm , J. Heiden , et al., “Antibodies to a Strain‐Specific Citrullinated Epstein–Barr Virus Peptide Diagnoses Rheumatoid Arthritis,” Scientific Reports 8, no. 1 (2018): 3684, 10.1038/s41598-018-22058-6. 29487382 PMC5829227

[9] J. Zheng , D. Baird , M. C. Borges , et al., “Recent Developments in Mendelian Randomization Studies,” Current Epidemiology Reports 4, no. 4 (2017): 330–345, 10.1007/s40471-017-0128-6. 29226067 PMC5711966

[10] J. Bowden , M. F. Del Greco , C. Minelli , G. Davey Smith , N. A. Sheehan , and J. R. Thompson , “Assessing the Suitability of Summary Data for Two‐Sample Mendelian Randomization Analyses Using MR‐Egger Regression: The Role of the I2 Statistic,” International Journal of Epidemiology 45, no. 6 (2016): 1961–1974, 10.1093/ije/dyw220. 27616674 PMC5446088

[11] M. Verbanck , C. Y. Chen , B. Neale , and R. Do , “Detection of Widespread Horizontal Pleiotropy in Causal Relationships Inferred From Mendelian Randomization Between Complex Traits and Diseases,” Nature Genetics 50, no. 5 (2018): 693–698, 10.1038/s41588-018-0099-7. 29686387 PMC6083837

[12] A. Perl , “Mechanisms of Viral Pathogenesis in Rheumatic Disease,” Annals of the Rheumatic Diseases 58, no. 8 (1999): 454–461, 10.1136/ard.58.8.454. 10419862 PMC1752929

[13] N. R. Jog and J. A. James , “Epstein Barr Virus and Autoimmune Responses in Systemic Lupus Erythematosus,” Frontiers in Immunology 11 (2021): 623944, 10.3389/fimmu.2020.623944. 33613559 PMC7886683

[14] X. Chen , H. Li , C. Wu , and Y. Zhang , “Epstein–Barr Virus and Human Herpesvirus 6 Infection in Patients With Systemic Lupus Erythematosus,” Virology Journal 20, no. 1 (2023): 29, 10.1186/s12985-023-01987-3. 36782252 PMC9926755

[15] M. Ramos‐Casals , M. J. Cuadrado , P. Alba , et al., “Acute Viral Infections in Patients With Systemic Lupus Erythematosus: Description of 23 Cases and Review of the Literature,” Medicine (Baltimore) 87, no. 6 (2008): 311–318, 10.1097/MD.0b013e31818ec711. 19011502

