# Supplementary material for: Exploring the Link Between Genetic Predictors of Systemic Lupus Erythematosus and Epstein–Barr Virus Infections
Source: Int J Rheum Dis. 2025 Feb 3;28(2):e70067. doi: 10.1111/1756-185X.70067 (PMC11790346; doi:10.1111/1756-185X.70067)
Supplement: Supplementary file 1 — Table S1. Table S2. Table S3. Table S4. Table S5. Table S6. Table S7. Table S8. [file APL-28-e70067-s001.docx]

**Supplementary Table 1.** Summary of GWAS data in this Mendelian randomization study

| **Trait** | **Source** | **Sample Size** | **case** | **control** | **ancestry** | **GWAS ID** |
| --- | --- | --- | --- | --- | --- | --- |
| Systemic lupus erythematosus (SLE) | Bentham J | 14267 | 5201 | 9066 | European | GCST003156 |
| Anti-Epstein-Barr virus (EBV) IgG levels | Chong A | 5010 | - | - | European | ieu-b-4901 |
| Anti-EBV viral capsid antigen (VCA) IgG levels | Scepanovic P | 956 | - | - | European | GCST006362 |
| Anti-EB-nuclear antigen (EBNA) IgG levels | Scepanovic P | 914 | - | - | European | GCST006361 |
| Anti-EBV-early antigen (EA) IgG levels | Scepanovic P | 91 | - | - | European | GCST006345 |
| infectious mononucleosis | FinnGen | 403953 | 2979 | 400974 | European | AB1_EBV |
| thyroid cancer | Kohler A | 1080 | 649 | 431 | European | ieu-a-1082 |
| breast cancer | Sakaue S | 257730 | 17389 | 240341 | European | GCST90018799 |
| malignant lymphoma | Sakaue S | 490803 | 3546 | 487257 | European | GCST90018878 |
| prostate cancer | Schumacher FR | 140254 | 79148 | 61106 | European | GCST006085 |
| endometrial cancer | O'Mara TA | 121885 | 12906 | 108979 | European | GCST006464 |
| gastic cancer | Sakaue S | 476116 | 1029 | 475087 | European | GCST90018849 |
| lung cancer | Sakaue S | 492803 | 3791 | 489012 | European | GCST90018875 |

| **Supplementary Table 2.** Detailed information of the instrument variables (IVs) in the forward Mendelian Randomization analysis | | | | | | | | | |
| --- | --- | --- | --- | --- | --- | --- | --- | --- | --- |
| **IVs for the effect of SLE in anti-EBV IgG** | | | | | | | | | |
| SNP | chr | pos | beta | se | samplesize | pval | eaf | effect_allele | other_allele |
| rs10048743 | 2 | 213890232 | 0.000528283 | 0.0326201 | NA | 0.98708 | 0.13968 | T | G |
| rs10200680 | 2 | 223961877 | 0.0501117 | 0.0318151 | NA | 0.115317 | 0.14718 | T | C |
| rs1078324 | 5 | 149202268 | 0.0568115 | 0.049399 | NA | 0.250192 | 0.053033 | A | C |
| rs10912578 | 1 | 173251856 | -0.0137972 | 0.0244804 | NA | 0.573057 | 0.32645 | G | A |
| rs1143679 | 16 | 31276811 | -0.0155435 | 0.0378156 | NA | 0.68107 | 0.10066 | A | G |
| rs12094036 | 1 | 183558174 | -0.0169868 | 0.0415719 | NA | 0.682846 | 0.079631 | C | T |
| rs12524498 | 6 | 31444187 | 0.0788555 | 0.0874769 | NA | 0.367408 | 0.017185 | T | G |
| rs13019891 | 2 | 113829869 | -0.0115771 | 0.022632 | NA | 0.609005 | 0.4559 | T | G |
| rs13136219 | 4 | 102743687 | 0.00403372 | 0.0236206 | NA | 0.864413 | 0.37962 | T | C |
| rs13332649 | 16 | 85966683 | 0.0253389 | 0.0271552 | NA | 0.350819 | 0.2224 | G | A |
| rs143123127 | 17 | 38009000 | 0.0311057 | 0.0605865 | NA | 0.607693 | 0.033276 | A | G |
| rs150180633 | 6 | 31015358 | -0.0307772 | 0.0993167 | NA | 0.756662 | 0.012328 | T | C |
| rs17849501 | 1 | 183542323 | -0.0756273 | 0.05681 | NA | 0.18319 | 0.045699 | T | C |
| rs2459611 | 2 | 191939499 | -0.00360151 | 0.0393709 | NA | 0.927119 | 0.905693 | T | C |
| rs2573219 | 2 | 233292383 | -0.085411 | 0.0392868 | NA | 0.0297625 | 0.092826 | C | A |
| rs268124 | 2 | 65654364 | -0.0273701 | 0.025701 | NA | 0.286968 | 0.26338 | T | C |
| rs34703115 | 2 | 40282854 | -0.139545 | 0.0735899 | NA | 0.0580003 | 0.027368 | C | T |
| rs35000415 | 7 | 128585616 | -0.0391007 | 0.0355608 | NA | 0.271598 | 0.11598 | T | C |
| rs35251378 | 19 | 10459969 | -0.0218781 | 0.0249436 | NA | 0.380484 | 0.29761 | A | G |
| rs353608 | 11 | 35101738 | -0.000386825 | 0.0225317 | NA | 0.986303 | 0.49075 | G | A |
| rs3747093 | 22 | 21985094 | -0.0553722 | 0.0300419 | NA | 0.0653808 | 0.19099 | A | G |
| rs389884 | 6 | 32035694 | -0.0439441 | 0.0334919 | NA | 0.18957 | 0.12933 | G | A |
| rs4274624 | 2 | 191958656 | -0.0123287 | 0.0267305 | NA | 0.644665 | 0.22933 | T | C |
| rs4388254 | 5 | 133428601 | 0.0610876 | 0.0584663 | NA | 0.296165 | 0.039417 | T | C |
| rs4661543 | 1 | 15231026 | 0.0220216 | 0.0341013 | NA | 0.518467 | 0.12438 | G | T |
| rs4916215 | 1 | 173314540 | -0.00365725 | 0.0251951 | NA | 0.884594 | 0.27384 | T | C |
| rs58688157 | 11 | 625085 | -0.0199181 | 0.0248846 | NA | 0.423516 | 0.27855 | G | A |
| rs58721818 | 6 | 138243739 | 0.0127457 | 0.063991 | NA | 0.842133 | 0.029625 | T | C |
| rs597808 | 12 | 111973358 | 0.0102882 | 0.0227472 | NA | 0.651088 | 0.4737 | G | A |
| rs6671847 | 1 | 161478810 | -0.0200842 | 0.0222311 | NA | 0.366355 | 0.49156 | A | G |
| rs6679677 | 1 | 114303808 | -0.0128057 | 0.0376706 | NA | 0.73392 | 0.10003 | A | C |
| rs6889239 | 5 | 150457771 | -0.0112899 | 0.0265392 | NA | 0.670567 | 0.23841 | C | T |
| rs7097397 | 10 | 50025396 | -0.00813536 | 0.023725 | NA | 0.731691 | 0.37309 | A | G |
| rs73050535 | 12 | 5012503 | -0.0815262 | 0.075236 | NA | 0.278607 | 0.023342 | T | C |
| rs73068668 | 19 | 55763262 | 0.0772942 | 0.044294 | NA | 0.0810588 | 0.07519 | A | G |
| rs7768653 | 6 | 106574794 | 0.0406628 | 0.023043 | NA | 0.0777016 | 0.39816 | T | C |
| rs7823055 | 8 | 55511676 | -0.0489325 | 0.0226576 | NA | 0.030861 | 0.43 | T | G |
| rs7899626 | 10 | 63825561 | -0.0241531 | 0.0248641 | NA | 0.331409 | 0.29738 | T | C |
| rs9852014 | 3 | 129084581 | -0.0676918 | 0.0448898 | NA | 0.131647 | 0.065527 | G | A |
| **IVs for the effect of SLE in EBV-VCA IgG** | | | | | | | | | |
| SNP | chr | pos | beta | se | samplesize | pval | eaf | effect_allele | other_allele |
| rs10048743 | 2 | 213890232 | 0.0062076 | 0.0311796 | 956 | 0.842234 | 0.859769 | T | G |
| rs10200680 | 2 | 223961877 | 0.0231987 | 0.0286228 | 956 | 0.417858 | 0.157038 | T | C |
| rs1078324 | 5 | 149203350 | -0.026472 | 0.0501263 | 956 | 0.59755 | 0.0504202 | A | C |
| rs10912578 | 1 | 173251856 | 0.0134548 | 0.0232641 | 956 | 0.563165 | 0.682773 | G | A |
| rs1143679 | 16 | 31276811 | -0.0285866 | 0.033809 | 956 | 0.398039 | 0.130507 | A | G |
| rs12094036 | 1 | 183558174 | -0.0285539 | 0.0374114 | 956 | 0.44551 | 0.0898109 | C | T |
| rs13019891 | 2 | 113829869 | -0.0143571 | 0.0220848 | 956 | 0.515792 | 0.482668 | T | G |
| rs13136219 | 4 | 102743687 | -0.0414422 | 0.0221269 | 956 | 0.0613861 | 0.372899 | T | C |
| rs13332649 | 16 | 85966683 | 0.0208425 | 0.0274608 | 956 | 0.448048 | 0.201681 | G | A |
| rs1464446 | 3 | 146601295 | -0.0559498 | 0.0275098 | 956 | 0.0422493 | 0.181723 | T | G |
| rs17849501 | 1 | 183542323 | -0.00790293 | 0.0499815 | 956 | 0.8744 | 0.0523128 | T | C |
| rs2431697 | 5 | 159879978 | 0.0132302 | 0.022142 | 956 | 0.550313 | 0.406388 | C | T |
| rs2459611 | 2 | 191939187 | -0.0434426 | 0.0327259 | 956 | 0.184675 | 0.879202 | T | C |
| rs2573219 | 2 | 233288667 | 0.036156 | 0.0374566 | 956 | 0.334654 | 0.0966387 | C | A |
| rs268124 | 2 | 65654364 | -0.00344741 | 0.024814 | 956 | 0.889535 | 0.742122 | T | C |
| rs35000415 | 7 | 128585616 | 0.0288241 | 0.0349149 | 956 | 0.409265 | 0.105042 | T | C |
| rs35251378 | 19 | 10459969 | -0.0320428 | 0.0235531 | 956 | 0.174013 | 0.294118 | A | G |
| rs353608 | 11 | 35101738 | -0.0228716 | 0.0218025 | 956 | 0.294431 | 0.516282 | G | A |
| rs3747093 | 22 | 21984379 | -0.0342387 | 0.0279096 | 956 | 0.220213 | 0.17437 | A | G |
| rs389884 | 6 | 31940897 | -0.0741884 | 0.0400896 | 956 | 0.0645595 | 0.0814978 | G | A |
| rs4274624 | 2 | 191958656 | 0.0060755 | 0.026293 | 956 | 0.817311 | 0.775735 | T | C |
| rs4661543 | 1 | 15229101 | -0.0243306 | 0.0314367 | 956 | 0.439151 | 0.86187 | G | T |
| rs4916215 | 1 | 173314540 | -0.00133203 | 0.0246381 | 956 | 0.956896 | 0.716912 | T | C |
| rs58688157 | 11 | 625085 | 0.00975305 | 0.0237571 | 956 | 0.68151 | 0.28729 | G | A |
| rs597808 | 12 | 111973358 | 0.0107767 | 0.0220273 | 956 | 0.624784 | 0.496324 | G | A |
| rs6671847 | 1 | 161478810 | -0.0197484 | 0.0210552 | 956 | 0.348519 | 0.47479 | A | G |
| rs6679677 | 1 | 114303808 | -0.0370357 | 0.0389899 | 956 | 0.342428 | 0.0919604 | A | C |
| rs6889239 | 5 | 150457771 | 0.0178897 | 0.0252158 | 956 | 0.47821 | 0.26208 | C | T |
| rs7097397 | 10 | 50025396 | 0.0158344 | 0.0231359 | 956 | 0.493894 | 0.395374 | A | G |
| rs73068668 | 19 | 55763262 | -0.0483214 | 0.0401683 | 956 | 0.229287 | 0.0761555 | A | G |
| rs7768653 | 6 | 106574794 | 0.00834494 | 0.0219374 | 956 | 0.703736 | 0.606092 | T | C |
| rs7823055 | 8 | 55511676 | -0.0187701 | 0.0218332 | 956 | 0.390171 | 0.565651 | T | G |
| rs7899626 | 10 | 63825561 | 0.0235169 | 0.0229606 | 956 | 0.305988 | 0.315126 | T | C |
| rs9852014 | 3 | 129084581 | 0.00851656 | 0.0427036 | 956 | 0.841966 | 0.0672269 | G | A |
| **IVs for the effect of SLE in EBNA IgG** | | | | | | | | | |
| SNP | chr | pos | beta | se | samplesize | pval | eaf | effect_allele | other_allele |
| rs10048743 | 2 | 213890232 | 0.0169962 | 0.0383144 | 914 | 0.657438 | 0.857849 | T | G |
| rs10200680 | 2 | 223961877 | -0.00792758 | 0.0349299 | 914 | 0.820508 | 0.160263 | T | C |
| rs1078324 | 5 | 149203350 | -0.0554448 | 0.0613304 | 914 | 0.366218 | 0.050494 | A | C |
| rs10912578 | 1 | 173251856 | -0.00531082 | 0.0285424 | 914 | 0.852434 | 0.68112 | G | A |
| rs1143679 | 16 | 31276811 | -0.0875393 | 0.0415617 | 914 | 0.0354715 | 0.12931 | A | G |
| rs12094036 | 1 | 183558174 | 0.0860174 | 0.0467407 | 914 | 0.0660511 | 0.0856202 | C | T |
| rs13019891 | 2 | 113829869 | 0.0108369 | 0.026897 | 914 | 0.687116 | 0.483535 | T | G |
| rs13136219 | 4 | 102743687 | 0.000947406 | 0.0271675 | 914 | 0.972189 | 0.372119 | T | C |
| rs13332649 | 16 | 85966683 | -0.0276229 | 0.0337057 | 914 | 0.412698 | 0.202525 | G | A |
| rs1464446 | 3 | 146601295 | -0.0268298 | 0.0337766 | 914 | 0.427212 | 0.182217 | T | G |
| rs17849501 | 1 | 183542323 | -0.108036 | 0.0606741 | 914 | 0.0753304 | 0.0528736 | T | C |
| rs2431697 | 5 | 159879978 | 0.0439814 | 0.0270499 | 914 | 0.10433 | 0.406322 | C | T |
| rs2459611 | 2 | 191939187 | -0.0680549 | 0.0409398 | 914 | 0.0967966 | 0.884742 | T | C |
| rs2573219 | 2 | 233288667 | 0.0260017 | 0.0461254 | 914 | 0.573086 | 0.0954995 | C | A |
| rs268124 | 2 | 65654364 | 0.0583383 | 0.0304371 | 914 | 0.0555943 | 0.744786 | T | C |
| rs35000415 | 7 | 128585616 | 0.0363845 | 0.0432871 | 914 | 0.400829 | 0.104281 | T | C |
| rs35251378 | 19 | 10459969 | -0.0133311 | 0.0289511 | 914 | 0.64529 | 0.291987 | A | G |
| rs353608 | 11 | 35101738 | -0.0120196 | 0.026857 | 914 | 0.654591 | 0.510977 | G | A |
| rs3747093 | 22 | 21984379 | 0.026035 | 0.0344986 | 914 | 0.450645 | 0.170692 | A | G |
| rs389884 | 6 | 31940897 | -0.0577154 | 0.0491927 | 914 | 0.241019 | 0.0821839 | G | A |
| rs4274624 | 2 | 191958656 | 0.0145438 | 0.0324063 | 914 | 0.653687 | 0.778266 | T | C |
| rs4661543 | 1 | 15229101 | -0.00771458 | 0.0385873 | 914 | 0.841584 | 0.862239 | G | T |
| rs4916215 | 1 | 173314540 | -0.0112705 | 0.030279 | 914 | 0.709815 | 0.719539 | T | C |
| rs58688157 | 11 | 625085 | 0.0252803 | 0.0293171 | 914 | 0.388748 | 0.289791 | G | A |
| rs597808 | 12 | 111973358 | 0.0213508 | 0.0271222 | 914 | 0.431367 | 0.492865 | G | A |
| rs6671847 | 1 | 161478810 | -0.0212845 | 0.0259362 | 914 | 0.412064 | 0.478046 | A | G |
| rs6679677 | 1 | 114303808 | -0.0579937 | 0.0469861 | 914 | 0.217437 | 0.0954023 | A | C |
| rs6889239 | 5 | 150457771 | 0.0502882 | 0.0309223 | 914 | 0.10424 | 0.262349 | C | T |
| rs7097397 | 10 | 50025396 | -0.0256504 | 0.0283119 | 914 | 0.365194 | 0.392529 | A | G |
| rs73068668 | 19 | 55763262 | -0.0133644 | 0.0500239 | 914 | 0.789405 | 0.0740944 | A | G |
| rs7768653 | 6 | 106574794 | -0.0471473 | 0.0268486 | 914 | 0.07942 | 0.604281 | T | C |
| rs7823055 | 8 | 55511676 | 0.0271969 | 0.0268425 | 914 | 0.311234 | 0.56202 | T | G |
| rs7899626 | 10 | 63825561 | -0.00587948 | 0.0283356 | 914 | 0.83567 | 0.310648 | T | C |
| rs9852014 | 3 | 129084581 | 0.0673363 | 0.0516985 | 914 | 0.193084 | 0.0691548 | G | A |
| **IVs for the effect of SLE in EBV-EA IgG** | | | | | | | | | |
| SNP | chr | pos | beta | se | samplesize | pval | eaf | effect_allele | other_allele |
| rs10048743 | 2 | 213890232 | -0.0289723 | 0.0777494 | 91 | 0.71038 | 0.780899 | T | G |
| rs10200680 | 2 | 223961877 | -0.0338806 | 0.0795997 | 91 | 0.671488 | 0.174157 | T | C |
| rs1078324 | 5 | 149203350 | 0.126301 | 0.15942 | 91 | 0.430499 | 0.0393258 | A | C |
| rs10912578 | 1 | 173251856 | -0.0536673 | 0.0736157 | 91 | 0.468067 | 0.758427 | G | A |
| rs1143679 | 16 | 31276811 | 0.0732762 | 0.0982846 | 91 | 0.458123 | 0.12069 | A | G |
| rs12094036 | 1 | 183558174 | 0.0471103 | 0.118179 | 91 | 0.691197 | 0.0730337 | C | T |
| rs13019891 | 2 | 113829869 | 0.0136933 | 0.0682627 | 91 | 0.841511 | 0.516854 | T | G |
| rs13136219 | 4 | 102743687 | 0.0476623 | 0.0634472 | 91 | 0.454675 | 0.325843 | T | C |
| rs13332649 | 16 | 85966683 | 0.0126862 | 0.0711211 | 91 | 0.858868 | 0.224719 | G | A |
| rs1464446 | 3 | 146601295 | -0.038471 | 0.0806695 | 91 | 0.634704 | 0.162921 | T | G |
| rs17849501 | 1 | 183542323 | -0.0189429 | 0.110079 | 91 | 0.863806 | 0.0747126 | T | C |
| rs2431697 | 5 | 159879978 | 0.0837493 | 0.0574886 | 91 | 0.149086 | 0.436782 | C | T |
| rs2459611 | 2 | 191939187 | -0.0693822 | 0.087448 | 91 | 0.429829 | 0.882022 | T | C |
| rs2573219 | 2 | 233288667 | -0.0894731 | 0.119229 | 91 | 0.455143 | 0.0730337 | C | A |
| rs268124 | 2 | 65654364 | 0.0658524 | 0.0662683 | 91 | 0.323281 | 0.741573 | T | C |
| rs35000415 | 7 | 128585616 | 0.0260317 | 0.0896997 | 91 | 0.772389 | 0.11236 | T | C |
| rs35251378 | 19 | 10459969 | -0.0444375 | 0.0697967 | 91 | 0.526113 | 0.280899 | A | G |
| rs353608 | 11 | 35101738 | 0.116151 | 0.0681407 | 91 | 0.0920598 | 0.511236 | G | A |
| rs3747093 | 22 | 21984379 | -0.0224963 | 0.0792064 | 91 | 0.777111 | 0.185393 | A | G |
| rs389884 | 6 | 31940897 | 0.174935 | 0.116187 | 91 | 0.136101 | 0.0632184 | G | A |
| rs4274624 | 2 | 191958656 | 0.101821 | 0.0702056 | 91 | 0.150784 | 0.735955 | T | C |
| rs4661543 | 1 | 15229101 | 0.0914685 | 0.097715 | 91 | 0.351983 | 0.893258 | G | T |
| rs4916215 | 1 | 173314540 | 0.0499474 | 0.0653839 | 91 | 0.447113 | 0.702247 | T | C |
| rs58688157 | 11 | 625085 | 0.0530518 | 0.0673239 | 91 | 0.432963 | 0.286517 | G | A |
| rs597808 | 12 | 111973358 | 0.0546006 | 0.066351 | 91 | 0.412948 | 0.404494 | G | A |
| rs6671847 | 1 | 161478810 | -0.0797927 | 0.0615543 | 91 | 0.198509 | 0.432584 | A | G |
| rs6679677 | 1 | 114303808 | -0.0735233 | 0.105247 | 91 | 0.486841 | 0.0862069 | A | C |
| rs6889239 | 5 | 150457771 | 0.106374 | 0.0752378 | 91 | 0.161194 | 0.230337 | C | T |
| rs7097397 | 10 | 50025396 | 0.0708534 | 0.0643444 | 91 | 0.27413 | 0.41954 | A | G |
| rs73068668 | 19 | 55763262 | -0.0773303 | 0.107227 | 91 | 0.472846 | 0.0955056 | A | G |
| rs7768653 | 6 | 106574794 | 0.000106803 | 0.0672234 | 91 | 0.998736 | 0.601124 | T | C |
| rs7823055 | 8 | 55511676 | 0.00394164 | 0.0608325 | 91 | 0.948495 | 0.634831 | T | G |
| rs7899626 | 10 | 63825561 | 0.0281387 | 0.0674326 | 91 | 0.677559 | 0.292135 | T | C |
| rs9852014 | 3 | 129084581 | 0.0111872 | 0.111537 | 91 | 0.92035 | 0.0730337 | G | A |
| **IVs for the effect of SLE in infectious mononucleosis** | | | | | | | | | |
| rs10048743 | 2 | 213890232 | -0.034 | 0.0532 | NA | 0.5221 | 0.817 | T | G |
| rs10200680 | 2 | 223961877 | -0.0532 | 0.0546 | NA | 0.3293 | 0.1688 | T | C |
| rs1078324 | 5 | 149202268 | 0.1267 | 0.1008 | NA | 0.2085 | 0.04163 | A | C |
| rs10912578 | 1 | 173251856 | -0.0126 | 0.044 | NA | 0.773701 | 0.6841 | G | A |
| rs1143679 | 16 | 31276811 | -0.027 | 0.064 | NA | 0.673701 | 0.1166 | A | G |
| rs12094036 | 1 | 183558174 | 0.0018 | 0.0713 | NA | 0.9798 | 0.09169 | C | T |
| rs12524498 | 6 | 31444187 | 0.1421 | 0.1461 | NA | 0.3307 | 0.01993 | T | G |
| rs13019891 | 2 | 113829869 | -0.0367 | 0.0412 | NA | 0.3735 | 0.4614 | T | G |
| rs13136219 | 4 | 102743687 | -0.0545 | 0.0426 | NA | 0.2006 | 0.3672 | T | C |
| rs13332649 | 16 | 85966683 | -0.0783 | 0.0455 | NA | 0.0856998 | 0.2769 | G | A |
| rs143123127 | 17 | 38009000 | -0.092 | 0.1008 | NA | 0.3617 | 0.04246 | A | G |
| rs1464446 | 3 | 146601295 | 0.0017 | 0.0537 | NA | 0.9748 | 0.1771 | T | G |
| rs150180633 | 6 | 31010047 | 0.0289 | 0.3442 | NA | 0.9331 | 0.003752 | T | C |
| rs17849501 | 1 | 183542323 | -0.0395 | 0.1065 | NA | 0.711001 | 0.03813 | T | C |
| rs2431697 | 5 | 159879978 | 0.0802 | 0.042 | NA | 0.0561798 | 0.3876 | C | T |
| rs2459611 | 2 | 191939187 | -0.1019 | 0.0913 | NA | 0.2644 | 0.9468 | T | C |
| rs2573219 | 2 | 233288667 | -0.0015 | 0.0734 | NA | 0.9833 | 0.0851 | C | A |
| rs268124 | 2 | 65654364 | 0.0183 | 0.0423 | NA | 0.6651 | 0.6249 | T | C |
| rs28361029 | 6 | 31220203 | 0.0963 | 0.0946 | NA | 0.3092 | 0.04927 | A | G |
| rs34703115 | 2 | 40282854 | 0.026 | 0.1149 | NA | 0.8206 | 0.03348 | C | T |
| rs35000415 | 7 | 128585616 | 0.1257 | 0.0571 | NA | 0.0277498 | 0.1503 | T | C |
| rs35251378 | 19 | 10459969 | 0.0236 | 0.0466 | NA | 0.611799 | 0.2615 | A | G |
| rs353608 | 11 | 35101738 | -0.0561 | 0.041 | NA | 0.1715 | 0.5336 | G | A |
| rs3747093 | 22 | 21984379 | 0.0563 | 0.0444 | NA | 0.2052 | 0.3126 | A | G |
| rs389884 | 6 | 31940897 | -0.0307 | 0.0761 | NA | 0.6868 | 0.09178 | G | A |
| rs4274624 | 2 | 191958656 | -0.03 | 0.0486 | NA | 0.5374 | 0.7688 | T | C |
| rs4388254 | 5 | 133428601 | 5.00E-04 | 0.0646 | NA | 0.9934 | 0.1127 | T | C |
| rs4661543 | 1 | 15229101 | -0.0558 | 0.0814 | NA | 0.4928 | 0.933 | G | T |
| rs4916215 | 1 | 173314540 | -0.0564 | 0.0527 | NA | 0.2846 | 0.8159 | T | C |
| rs58688157 | 11 | 625085 | 0.0044 | 0.0506 | NA | 0.9312 | 0.2063 | G | A |
| rs58721818 | 6 | 138243739 | 0.3288 | 0.1545 | NA | 0.0333304 | 0.01805 | T | C |
| rs597808 | 12 | 111973358 | -0.0415 | 0.0415 | NA | 0.3176 | 0.5854 | G | A |
| rs6671847 | 1 | 161478810 | -0.0134 | 0.0412 | NA | 0.7456 | 0.4542 | A | G |
| rs6679677 | 1 | 114303808 | -0.0646 | 0.0573 | NA | 0.2598 | 0.1472 | A | C |
| rs6889239 | 5 | 150457771 | 0.0511 | 0.0454 | NA | 0.2604 | 0.2865 | C | T |
| rs7097397 | 10 | 50025396 | 0.0087 | 0.0427 | NA | 0.8389 | 0.3697 | A | G |
| rs73050535 | 12 | 5012503 | -0.3356 | 0.2872 | NA | 0.2425 | 0.005013 | T | C |
| rs73068668 | 19 | 55763262 | -0.0056 | 0.0745 | NA | 0.9405 | 0.08297 | A | G |
| rs7768653 | 6 | 106574794 | -0.045 | 0.0416 | NA | 0.2797 | 0.5333 | T | C |
| rs7823055 | 8 | 55511676 | -0.0107 | 0.0412 | NA | 0.7957 | 0.5686 | T | G |
| rs7899626 | 10 | 63825561 | 0.0509 | 0.0423 | NA | 0.2287 | 0.3923 | T | C |
| rs9852014 | 3 | 129084581 | -0.0497 | 0.0756 | NA | 0.5108 | 0.07964 | G | A |

| **Supplementary Table 3.** Detailed information of the instrument variables (IVs) in the reverse Mendelian Randomization analysis | | | | | | | | | |
| --- | --- | --- | --- | --- | --- | --- | --- | --- | --- |
| **IVs for the effect of anti-EBV IgG in SLE** | | | | | | | | | |
| SNP | chr | pos | beta | se | samplesize | pval | eaf | effect_allele | other_allele |
| rs1038194 | 9 | 137855579 | -0.131028 | 0.0951696 | 14267 | 0.168578 | NA | G | A |
| rs10515742 | 5 | 156155832 | 0.0295588 | 0.0700007 | 14267 | 0.672833 | NA | G | A |
| rs13140733 | 4 | 2920937 | 0.0198026 | 0.0261861 | 14267 | 0.449514 | NA | A | G |
| rs138422303 | 9 | 101754323 | -0.0202027 | 0.0760104 | 14267 | 0.790402 | NA | A | G |
| rs138452122 | 1 | 212592575 | 0.207014 | 0.125238 | 14267 | 0.0983354 | NA | A | G |
| rs144794356 | 3 | 26424204 | -0.198451 | 0.144572 | 14267 | 0.169852 | NA | C | T |
| rs1856619 | 1 | 100019409 | 0.0198026 | 0.030596 | 14267 | 0.517483 | NA | A | G |
| rs2504818 | 6 | 22491695 | -0.00995033 | 0.0352172 | 14267 | 0.777528 | NA | A | G |
| rs3096702 | 6 | 32192331 | 0.0833816 | 0.0334619 | 14267 | 0.0127084 | NA | G | A |
| rs4129254 | 11 | 101365032 | 0.0198026 | 0.0374437 | 14267 | 0.596901 | NA | G | A |
| rs4346749 | 5 | 10860334 | -0.0304592 | 0.0414614 | 14267 | 0.462559 | NA | A | G |
| rs61251820 | 3 | 150063821 | -0.0100503 | 0.0517507 | 14267 | 0.846014 | NA | T | C |
| rs62100736 | 19 | 34054649 | -0.040822 | 0.0447044 | 14267 | 0.361162 | NA | C | A |
| rs62131109 | 2 | 34371502 | 0.00995033 | 0.0489562 | 14267 | 0.83894 | NA | G | A |
| rs72674728 | 4 | 125024289 | 0.0392207 | 0.0551451 | 14267 | 0.476942 | NA | T | C |
| rs73235908 | 3 | 113915496 | -0.18633 | 0.121697 | 14267 | 0.125745 | NA | T | C |
| rs74807157 | 10 | 62076002 | -0.0512933 | 0.0911706 | 14267 | 0.573702 | NA | A | C |
| rs75285142 | 5 | 73598469 | -0.0618754 | 0.140751 | 14267 | 0.66022 | NA | C | T |
| **IVs for the effect of EBV-VCA IgG in SLE** | | | | | | | | | |
| SNP | chr | pos | beta | se | samplesize | pval | eaf | effect_allele | other_allele |
| rs1662823 | 18 | 3311748 | 0.0100503 | 0.0271013 | 14267 | 0.710753 | NA | T | G |
| rs2163916 | 17 | 65534543 | 0.00995033 | 0.070991 | 14267 | 0.888531 | NA | A | G |
| rs245064 | 5 | 149332996 | -0.0304592 | 0.033858 | 14267 | 0.368324 | NA | T | C |
| rs6556882 | 5 | 95138353 | 0.00995033 | 0.0216182 | 14267 | 0.645318 | NA | C | T |
| rs6985207 | 8 | 8604423 | -0.105361 | 0.0280466 | 14267 | 0.000172219 | NA | C | A |
| rs9876198 | 3 | 132685783 | 0.0198026 | 0.029956 | 14267 | 0.508575 | NA | T | C |
| **IVs for the effect of EBNA IgG in SLE** | | | | | | | | | |
| SNP | chr | pos | beta | se | samplesize | pval | eaf | effect_allele | other_allele |
| rs10226349 | 7 | 17710866 | -0.0100503 | 0.0770142 | 14267 | 0.896171 | NA | T | C |
| rs1714631 | 6 | 107304447 | -0.00995033 | 0.0406815 | 14267 | 0.806773 | NA | C | T |
| rs17452718 | 7 | 111601681 | -0.0202027 | 0.045227 | 14267 | 0.655095 | NA | G | T |
| rs4555924 | 6 | 150259790 | 0.0295588 | 0.0387033 | 14267 | 0.445029 | NA | G | A |
| rs530411 | 19 | 39927540 | 0.00995033 | 0.0223101 | 14267 | 0.655596 | NA | T | C |
| rs59217282 | 21 | 22074639 | -0.0618754 | 0.052473 | 14267 | 0.238324 | NA | T | C |
| rs6895504 | 5 | 109612768 | 0.0392207 | 0.0390108 | 14267 | 0.314714 | NA | C | T |
| rs6927022 | 6 | 32612397 | 0.239017 | 0.0306765 | 14267 | 6.62E-15 | NA | G | A |
| rs72738076 | 5 | 18660257 | 0.0676586 | 0.0567789 | 14267 | 0.233412 | NA | T | C |
| rs77272118 | 6 | 70583157 | -0.0304592 | 0.0483235 | 14267 | 0.528485 | NA | T | C |
| **IVs for the effect of EBV-EA IgG in SLE** | | | | | | | | | |
| SNP | chr | pos | beta | se | samplesize | pval | eaf | effect_allele | other_allele |
| rs13107682 | 4 | 166894882 | 0.0100503 | 0.0176459 | 14267 | 0.568977 | NA | G | A |
| rs1457795 | 3 | 164601497 | -0.0198026 | 0.0362723 | 14267 | 0.585105 | NA | C | T |
| rs34461918 | 5 | 83028301 | -0.0833816 | 0.0549116 | 14267 | 0.128896 | NA | C | A |
| rs61277217 | 17 | 76492840 | 0.0582689 | 0.0293745 | 14267 | 0.0472944 | NA | G | A |
| rs72780318 | 5 | 117931616 | -0.0304592 | 0.0371454 | 14267 | 0.412216 | NA | G | A |
| rs8081555 | 17 | 14889595 | 0.0198026 | 0.0453405 | 14267 | 0.66229 | NA | C | T |
| rs874628 | 19 | 18304700 | -0.0725707 | 0.0321514 | 14267 | 0.0239983 | NA | G | A |
| **IVs for the effect of infectious mononucleosis in SLE** | | | | | | | | | |
| rs11265380 | 1 | 160348727 | -0.0833816 | 0.028559 | 14267 | 0.00350445 | NA | A | G |
| rs112824579 | 2 | 242550410 | 0.122218 | 0.0411632 | 14267 | 0.00298676 | NA | A | C |
| rs116856714 | 9 | 12052324 | -0.0618754 | 0.105921 | 14267 | 0.559108 | NA | T | C |
| rs12599876 | 16 | 68393405 | 0.0582689 | 0.0497169 | 14267 | 0.241191 | NA | T | C |
| rs12654043 | 5 | 150226095 | -0.0304592 | 0.0433523 | 14267 | 0.482307 | NA | G | A |
| rs143465656 | 3 | 34716743 | -0.105361 | 0.0764983 | 14267 | 0.168422 | NA | G | T |
| rs145215254 | 2 | 117365074 | 0.0676586 | 0.102657 | 14267 | 0.509846 | NA | T | C |
| rs149485602 | 9 | 87522592 | -0.0100503 | 0.150316 | 14267 | 0.946692 | NA | C | T |
| rs17160581 | 7 | 138541212 | -0.0100503 | 0.0447563 | 14267 | 0.822324 | NA | T | C |
| rs1773231 | 3 | 194392683 | -0.00995033 | 0.0492327 | 14267 | 0.839832 | NA | C | A |
| rs188985267 | 17 | 14374754 | 0.182322 | 0.132736 | 14267 | 0.169578 | NA | A | G |
| rs2518778 | 7 | 90797314 | 0.0512933 | 0.0703502 | 14267 | 0.465932 | NA | C | T |
| rs2615200 | 18 | 60961675 | 0.0304592 | 0.0273521 | 14267 | 0.265453 | NA | T | G |
| rs34242269 | 8 | 144207738 | 0.0487902 | 0.0302832 | 14267 | 0.107151 | NA | T | G |
| rs36173887 | 6 | 32602086 | -0.248461 | 0.0608538 | 14267 | 4.45E-05 | NA | G | A |
| rs45611335 | 16 | 88598489 | 0.0295588 | 0.0437781 | 14267 | 0.499551 | NA | T | C |
| rs60492066 | 4 | 177051633 | 0.0676586 | 0.0318851 | 14267 | 0.0338423 | NA | C | T |
| rs72649979 | 1 | 212018435 | -0.0725707 | 0.0720182 | 14267 | 0.313612 | NA | G | A |
| rs73171666 | 3 | 153472977 | 0.0295588 | 0.0469742 | 14267 | 0.529181 | NA | G | T |
| rs73203224 | 7 | 110080785 | -0.18633 | 0.128335 | 14267 | 0.14653 | NA | C | T |
| rs76115077 | 6 | 72453607 | -0.105361 | 0.110823 | 14267 | 0.341752 | NA | G | A |
| rs76330758 | 1 | 175233273 | -0.127833 | 0.0727 | 14267 | 0.0786846 | NA | G | A |
| rs76635736 | 6 | 84454084 | 0.00995033 | 0.0586788 | 14267 | 0.865346 | NA | A | G |
| rs77257060 | 3 | 184452890 | -0.0833816 | 0.0711589 | 14267 | 0.241291 | NA | A | G |
| rs79612296 | 17 | 62504233 | -0.0202027 | 0.099834 | 14267 | 0.839633 | NA | A | G |
| rs9922412 | 16 | 28347707 | 0.0392207 | 0.0317677 | 14267 | 0.216976 | NA | T | C |

**Supplementary Table 4.** MR estimates from each method of assessing the causal effects of SLE on EBV.

(Note: EBV=Epstein-Barr virus; SLE=Systemic lupus erythematosus; VCA=viral capsid antigen; EBNA=Epstein-Barr virus nuclear antigen; EA=early antigen; IVW=inverse vatiance weighted; WM=weighted median)

| **MR-Method** | | **SLE (Exposure)** | | | |
| --- | --- | --- | --- | --- | --- |
|  |  | **Number of SNPs** | **OR(95% CI)** | **SE** | **p-value** |
| anti-EBV IgG | IVW | 39 | 0.973(0.949-1.002) | 0.014 | 0.074 |
|  | WM | 39 | 0.965(0.926-1.006) | 0.021 | 0.091 |
|  | MR-Egger | 39 | 0.978(0.924-1.034) | 0.029 | 0.434 |
|  | simple mode | 39 | 0.971(0.895-1.053) | 0.044 | 0.511 |
|  | weighted mode | 39 | 0.968(0.914-1.025) | 0.026 | 0.223 |
| EBV-VCA IgG | IVW | 34 | 0.998(0.971-1.025) | 0.014 | 0.863 |
|  | WM | 34 | 0.990(0.951-1.031) | 0.020 | 0.664 |
|  | MR-Egger | 34 | 1.001(0.945-1.059) | 0.029 | 0.983 |
|  | simple mode | 34 | 0.947(0.880-1.017) | 0.036 | 0.146 |
|  | weighted mode | 34 | 1.006(0.949-1.065) | 0.031 | 0.853 |
| EBNA IgG | IVW | 34 | 0.984(0.949-1.019) | 0.018 | 0.358 |
|  | WM | 34 | 0.997(0.930-1.026) | 0.026 | 0.350 |
|  | MR-Egger | 34 | 0.961(0.891-1.037) | 0.039 | 0.310 |
|  | simple mode | 34 | 0.992(0.894-1.102) | 0.052 | 0.885 |
|  | weighted mode | 34 | 0.973(0.910-1.040) | 0.033 | 0.442 |
| EBV-EA IgG | IVW | 34 | 1.000(0.927-1.077) | 0.038 | 0.993 |
|  | WM | 34 | 0.983(0.881-1.097) | 0.053 | 0.763 |
|  | MR-Egger | 34 | 1.017(0.868-1.193) | 0.081 | 0.833 |
|  | simple mode | 34 | 1.087(0.888-1.330) | 0.099 | 0.426 |
|  | weighted mode | 34 | 1.001(0.863-1.160) | 0.079 | 0.993 |
| infectious mononucleosis | IVW | 42 | 1.020(0.973-1.070) | 0.024 | 0.401 |
|  | WM | 42 | 1.002(0.934-1.075) | 0.036 | 0.955 |
|  | MR-Egger | 42 | 0.998(0.900-1.106) | 0.053 | 0.967 |
|  | simple mode | 42 | 0.990(0.873-1.122) | 0.064 | 0.875 |
|  | weighted mode | 42 | 1.003(0.915-1.101) | 0.047 | 0.937 |

**Supplementary Table 5.** MR estimates from each method of assessing the causal effects of EBV on SLE.

| **MR-Method** | | **SLE (Outcome)** | | | |
| --- | --- | --- | --- | --- | --- |
|  |  | **Number of SNPs** | **OR(95% CI)** | **SE** | **p-value** |
| anti-EBV IgG | IVW | 18 | 0.982(0.858-1.124) | 0.069 | 0.789 |
|  | WM | 18 | 0.987(0.826-1.178) | 0.091 | 0.883 |
|  | MR-Egger | 18 | 1.136(0.814-1.585) | 0.170 | 0.463 |
|  | simple mode | 18 | 0.961(0.685-1.349) | 0.173 | 0.822 |
|  | weighted mode | 18 | 1.058(0.780-1.435) | 0.155 | 0.719 |
| EBV-VCA IgG | IVW | 6 | 1.181(0.821-1.689) | 0.185 | 0.370 |
|  | WM | 6 | 0.919(0.683-1.238) | 0.166 | 0.573 |
|  | MR-Egger | 6 | 0.005(4.439e-07-56.570) | 4.761 | 0.328 |
|  | simple mode | 6 | 0.918(0.630-1.338) | 0.206 | 0.702 |
|  | weighted mode | 6 | 0.918(0.688-1.227) | 0.162 | 0.617 |
| EBNA IgG | IVW | 10 | 0.841(0.578-1.224) | 0.191 | 0.367 |
|  | WM | 10 | 1.101(0.891-1.361) | 0.108 | 0.372 |
|  | MR-Egger | 10 | 0.464(0.043-5.053) | 1.218 | 0.546 |
|  | simple mode | 10 | 1.150(0.875-1.511) | 0.130 | 0.342 |
|  | weighted mode | 10 | 1.133(0.892-1.439) | 0.120 | 0.331 |
| EBV-EA IgG | IVW | 7 | 0.981(0.879-1.095) | 0.056 | 0.734 |
|  | WM | 7 | 1.013(0.910-1.128) | 0.055 | 0.814 |
|  | MR-Egger | 7 | 0.845(0.495-1.442) | 0.273 | 0.563 |
|  | simple mode | 7 | 0.916(0.744-1.128) | 0.105 | 0.441 |
|  | weighted mode | 7 | 1.021(0.892-1.169) | 0.066 | 0.770 |
| infectious mononucleosis | IVW | 26 | 0.972(0.908-1.040) | 0.035 | 0.407 |
|  | WM | 26 | 0.984(0.920-1.054) | 0.035 | 0.649 |
|  | MR-Egger | 26 | 0.976(0.874-1.090) | 0.056 | 0.675 |
|  | simple mode | 26 | 0.899(0.803-1.006) | 0.057 | 0.076 |
|  | weighted mode | 26 | 0.974(0.911-1.041) | 0.034 | 0.443 |

(Note: EBV=Epstein-Barr virus; SLE=systemic lupus erythematosus; VCA=viral capsid antigen; EBNA=Epstein-Barr virus nuclear antigen; EA=early antigen; IVW=inverse vatiance weighted; WM=weighted median)

| **Exposure** | **Outcome** | **Pleiotropy test** | | |  | **Heterogeneity test** | | | | | | |
| --- | --- | --- | --- | --- | --- | --- | --- | --- | --- | --- | --- | --- |
|  |  | **MR-Egger** | | |  | **MR-Egger** | | |  | **Inverse vatiance weighted** | | |
|  |  | **Intercept** | **SE** | **P** |  | **Q** | **Q_df** | **Q_pval** |  | **Q** | **Q_df** | **Q_pval** |
| **SLE** | **EBV IgG** | -0.001 | 0.011 | 0.930 |  | 40.676 | 37 | 0.308 |  | 40.775 | 38 | 0.349 |
|  | **VCA IgG** | -0.001 | 0.010 | 0.908 |  | 30.101 | 32 | 0.563 |  | 30.115 | 33 | 0.611 |
|  | **EBNA IgG** | 0.009 | 0.013 | 0.499 |  | 37.816 | 32 | 0.221 |  | 38.368 | 33 | 0.239 |
|  | **EA IgG** | -0.001 | 0.028 | 0.807 |  | 24.022 | 32 | 0.844 |  | 24.082 | 33 | 0.871 |
|  | **infectious mononucleosis** | 0.009 | 0.081 | 0.631 |  | 39.195 | 40 | 0.506 |  | 39.430 | 41 | 0.541 |

**Supplementary Table 6.** Pleiotropy and Heterogeneity test of the SLE IVs from EBV infection GWAS

**Supplementary Table 7.** Pleiotropy and Heterogeneity test of the EBV infection IVs from SLE GWAS

| **Outcome** | **Exposure** | **Pleiotropy test** | | |  | **Heterogeneity test** | | | | | | |
| --- | --- | --- | --- | --- | --- | --- | --- | --- | --- | --- | --- | --- |
|  |  | **MR-Egger** | | |  | **MR-Egger** | | |  | **Inverse vatiance weighted** | | |
|  |  | **Intercept** | **SE** | **P** |  | **Q** | **Q_df** | **Q_pval** |  | **Q** | **Q_df** | **Q_pval** |
| **SLE** | **EBV IgG** | -0.027 | 0.029 | 0.361 |  | 18.053 | 16 | 0.321 |  | 19.053 | 17 | 0.326 |
|  | **VCA IgG** | 0.582 | 0.507 | 0.315 |  | 10.189 | 4 | 0.037 |  | 13.547 | 5 | 0.019 |
|  | **EBNA IgG** | 0.099 | 0.201 | 0.634 |  | 58.703 | 8 | 8.37e-10 |  | 60.504 | 9 | 1.07e-09 |
|  | **EA IgG** | 0.046 | 0.081 | 0.598 |  | 11.828 | 5 | 0.037 |  | 12.578 | 6 | 0.050 |
|  | **infectious mononucleosis** | -0.003 | 0.024 | 0.914 |  | 58.699 | 24 | 0.000 |  | 58.728 | 25 | 0.000 |

| **Supplementary Table 8.** Estimated proportion of the effect of SLE on cancers mediated by EBV infections | | | | | | | | |
| --- | --- | --- | --- | --- | --- | --- | --- | --- |
| Exposure | Mediator | Outcome | β_all | βa | βb | β_dir | Mediation Effect | |
|  |  |  |  |  |  |  | βc(95%CI) | P-val |
| SLE | thyroid cancer | Anti-EBV IgG | -0.025 | 0.806 | 4.51e-05 | -0.025 | 3.63e-05  (-0.281, 0.281) | 1.000 |
|  |  | Anti-VCA IgG | -0.002 | 0.806 | 0.001 | -0.003 | 0.001  (-0.281, 0.282) | 0.998 |
|  |  | Anti-EBNA IgG | -0.017 | 0.806 | -0.000 | -0.016 | -0.000  (-0.282, 0.281) | 0.999 |
|  |  | Anti-EA IgG | -0.000 | 0.806 | 0.000 | -0.001 | 0.000  (-0.281, 0.282) | 0.999 |
|  |  | infectious mononucleosis | 0.020 | 0.806 | 0.003 | 0.018 | 0.002  (-0.279, 0.283) | 0.994 |
| SLE | breast cancer | Anti-EBV IgG | -0.025 | -0.002 | 0.005 | -0.025 | -1.14e-05  (-0.0002, 0.0002) | 0.953 |
|  |  | Anti-VCA IgG | -0.002 | -0.002 | -0.041 | -0.002 | 9.66e-05  (-0.002, 0.002) | 0.956 |
|  |  | Anti-EBNA IgG | -0.017 | -0.002 | 0.031 | -0.016 | -7.45e-05  (-0.002, 0.001) | 0.959 |
|  |  | Anti-EA IgG | -0.000 | -0.002 | -0.085 | -0.001 | 0.000  (-0.009, 0.009) | 0.982 |
|  |  | infectious mononucleosis | 0.020 | -0.002 | -0.120 | 0.020 | 0.000  (-0.008, 0.008) | 0.972 |
| SLE | malignant lymphoma | Anti-EBV IgG | -0.025 | 0.025 | 0.069 | -0.027 | 0.002  (-0.005, 0.008) | 0.790 |
|  |  | Anti-VCA IgG | -0.002 | 0.025 | -0.029 | -0.002 | -0.001  (-0.004, 0.002) | 0.803 |
|  |  | Anti-EBNA IgG | -0.017 | 0.025 | 0.027 | -0.017 | 0.001  (-0.006, 0.007) | 0.916 |
|  |  | Anti-EA IgG | -0.000 | 0.025 | 0.198 | -0.005 | 0.005  (-0.034, 0.044) | 0.899 |
|  |  | infectious mononucleosis | 0.024 | 0.025 | -0.156 | 0.024 | -0.004  (-0.039, 0.031) | 0.911 |
| SLE | prostate cancer | Anti-EBV IgG | -0.245 | -0.011 | -0.013 | -0.025 | 0.000  (-0.0002, 0.0004) | 0.715 |
|  |  | Anti-VCA IgG | -0.002 | -0.011 | 0.010 | -0.002 | -0.000  (-0.0004, 0.0002) | 0.712 |
|  |  | Anti-EBNA IgG | -0.017 | -0.011 | 0.043 | -0.016 | -0.000  (-0.002, 0.001) | 0.751 |
|  |  | Anti-EA IgG | -0.000 | -0.011 | -0.050 | -0.001 | 0.001  (-0.003, 0.004) | 0.889 |
|  |  | infectious mononucleosis | 0.020 | -0.011 | -0.055 | 0.020 | 0.001  (-0.002, 0.003) | 0.815 |
| SLE | endometrial cancer | Anti-EBV IgG | -0.025 | -0.040 | 0.022 | -0.024 | -0.001  (-0.002, 0.001) | 0.553 |
|  |  | Anti-VCA IgG | -0.002 | -0.040 | 0.072 | 0.001 | -0.003  (-0.008, 0.002) | 0.563 |
|  |  | Anti-EBNA IgG | -0.017 | -0.040 | -0.026 | -0.018 | 0.001  (-0.001, 0.003) | 0.606 |
|  |  | Anti-EA IgG | -0.000 | -0.040 | -0.112 | -0.005 | 0.005  (-0.014, 0.024) | 0.813 |
|  |  | infectious mononucleosis | 0.020 | -0.040 | -0.145 | 0.015 | 0.006  (-0.009, 0.021) | 0.700 |
| SLE | gastic cancer | Anti-EBV IgG | -0.025 | -0.001 | -0.242 | -0.025 | 0.000  (-0.028, 0.029) | 0.994 |
|  |  | Anti-VCA IgG | -0.002 | -0.001 | 0.068 | -0.002 | -6.23e-05  (-0.007, 0.007) | 0.993 |
|  |  | Anti-EBNA IgG | -0.017 | -0.001 | -0.025 | -0.017 | 2.25e-05  (-0.003, 0.003) | 0.994 |
|  |  | Anti-EA IgG | -0.000 | -0.001 | -0.151 | -0.000 | 1.38e-04  (-0.042, 0.042) | 0.997 |
|  |  | infectious mononucleosis | 0.020 | -0.001 | -0.003 | 0.020 | 3.20e-06  (-0.000, 0.000) | 0.996 |
| SLE | lung cancer | Anti-EBV IgG | -0.025 | 0.014 | -0.045 | -0.024 | -0.001  (-0.006, 0.005) | 0.916 |
|  |  | Anti-VCA IgG | -0.002 | 0.014 | -0.076 | -0.001 | -0.001  (-0.006, 0.004) | 0.845 |
|  |  | Anti-EBNA IgG | -0.017 | 0.014 | 0.036 | -0.017 | 0.000  (-0.003, 0.003) | 0.874 |
|  |  | Anti-EA IgG | -0.000 | 0.014 | -0.265 | 0.003 | -0.003  (-0.054, 0.047) | 0.942 |
|  |  | infectious mononucleosis | 0.020 | 0.014 | -0.114 | 0.022 | -0.002  (-0.017, 0.014) | 0.917 |
